# LncRNA FTX represses the progression of non-alcoholic fatty liver disease to hepatocellular carcinoma via regulating the M1/M2 polarization of Kupffer cells

**DOI:** 10.1186/s12935-020-01354-0

**Published:** 2020-06-24

**Authors:** Huajun Wu, Zhiwei Zhong, Anji Wang, Chunhui Yuan, Ke Ning, Huanhuan Hu, Chao Wang, Xiangbao Yin

**Affiliations:** 1 Department of Hepatobiliary and Pancreatic Surgery, The Second Affiliated Hospital of Nanchang University, No. 1 Minde Road, Nanchang, Jiangxi Province China; 2 Department of Vascular Surgery, The Second Affiliated Hospital of Nanchang University, Nanchang, Jiangxi Province China

**Keywords:** Hepatocellular carcinoma, lncRNA FTX, Non-alcoholic fatty liver disease, Kupffer cell

## Abstract

**Background:**

The effect of lncRNA FTX on non-alcoholic fatty liver disease (NAFLD) conversion to hepatocellular carcinoma (HCC) is unclear.

**Methods:**

In our study, C57BL/6 mice was fed with high fat diet for obtaining NAFLD mouse model, and diethylnitrosamine induced the formation of HCC tumor. The expression of iNOS and CD206 in tissues were examined using immunohistochemistry. In addition, qRT-PCR was implemented to detect the expression of FTX and mRNAs. The percentage of M1 and M2 Kupffer cells (KCs) were determined using flow cytometry. The pathological change in liver tissues was displayed by H&E staining. Besides, immunofluorescence assay was performed to ensure the primary KCs through labeling F4/80.

**Results:**

Here, we found that the expression of FTX and the ratio of M1/M2 KCs in liver tissues from NAFLD-transformed HCC (NAFLD-HCC) patients lower than in liver tissues from NAFLD patients. Subsequently, we revealed that the expression of FTX and M1/M2 KCs ratio were downregulated during NAFLD conversion to HCC. Importantly, increasing of FTX inhibited HCC tumor growth, improved liver damage and promoted M1 polarization of KCs during NAFLD conversion to HCC, while these effects of FTX were reversed by inactivating of KCs. Finally, in vitro experiments, our data indicated that FTX facilitated the M1 polarization of KCs.

**Conclusion:**

In conclusion, our results demonstrated that upregulation of FTX suppressed NAFLD conversion to HCC though promoting M1 polarization of KCs. Our findings presented a new regulatory mechanism for NAFLD conversion to HCC, and provided a new biomarker for inhibiting this conversion.

## Background

Hepatocellular carcinoma (HCC) is the fifth most common cancer in the world, and about one million new cases of HCC were diagnosed annually [1]. Cirrhosis is the primary risk factor for HCC. In China, hepatitis B virus, as one of the most common reason in cirrhosis, has been considered as the major contributor to HCC [2]. Whereas, with the dissemination of anti-viral treatment, the changes of HCC causer is ongoing. Increasing evidence have demonstrated that the number of patients with HCC induced by non-alcoholic fatty liver disease (NAFLD) is increasing, NAFLD is expected to become a leading reason of the incidence and mortality in HCC [3]. However, the underlying molecular mechanism of NAFLD conversion to HCC remains unclear.

Macrophage involves in the progression of many malignant tumors via acting as a major component of innate cellular immunity [4]. It has been known that according to the stimulation of microenvironmental, macrophage can differentiate into two types: classically activated M1 macrophage and alternatively activated M2 macrophage, which have the opposite function in inflammatory responses [5]. M1 macrophage promotes the progression of inflammation through secreting pro-inflammatory cytokines, such as TNF-α and IL-1β, while M2 macrophage is anti-inflammatory via inducing lowly expressed IL-12 and highly expressed of IL-10 and TGFβ [6]. Importantly, it has been indicated that M1 macrophage represses the progression of cancer, while M2 macrophage promotes the progression of cancer [7]. Kupffer cell (KC) is the biggest macrophage as well as the sessile resident live macrophage [8]. It has been reported that the cell number of KCs in HCC tissues is lower than matched normal tissues, and KCs could effectively repress the proliferation of HCC cell lines [9]. In addition, Wang et al. proposed that the M1/M2 ratio in KCs is decreased in NAFLD-transformed HCC (NALFD-HCC) patients, and increasing M1 polarization of KCs remarkably inhibits the development of NAFLD to HCC [10]. However, the change of KCs polarization and accurate role of KCs in the progression of NAFLD to HCC remain unclear.

Long non-coding RNAs (lncRNAs) belong to non-coding RNA with more than 200 nucleotides in length. A growing reliable evidence demonstrated that lncRNAs are associated with the progression of multiple malignant tumors including HCC [11]. Such as, it has been indicated that lncRNA PARP1 is highly expressed in HCC tissues, and facilitates the progression of HCC via increasing the expression of PARP1 [12]. While, another lncRNA, NKILA, has been proved to be decreased in HCC tissues, and increasing the expression of NKILA significantly enhances the anti-cancer effects of baicalein in HCC though targeting to its downstream targets [13]. Furthermore, a series of lncRNAs have been also indicated to aberrantly express in NAFLD, and these lncRNAs may be linked with the development of NAFLD [14]. A recent research has shown that the lncRNA FTX is upregulated in liver tissues from high fat diet (HFD)-induced NAFLD mouse [15]. However, the effect of FTX on NAFLD progression is unclear. Interestingly, Liu et al. proved that FTX is downregulated in HCC tissues, and increasing the expression of FTX could repress HCC progression via targeting to minichromosome maintenance protein 2 and miR-347a [16].

In this present study, we investigated the effect of FTX on KCs polarization and the role of it in the progression of NAFLD to HCC. Here, we found that FTX was downregulated in NAFLD-HCC tumors tissues, and upregulation of FTX suppressed the development of NAFLD to HCC via promoting M1 polarization of KCs. Our data indicated that FTX maybe a novel biomarker for NAFLD-HCC treatment.

## Materials and methods

### Clinical samples

Fresh liver tissues in 20 patients with NAFLD, and 25 cases of NAFLD-HCC and matched paracancerous tissues were obtained from The Second Affiliated Hospital of Nanchang University, and frozen in liquid nitrogen until to use in next experiments. All patients signed the informed consent, and all studies were accomplished in accordance with the guidelines of pathological specimen handing. Moreover, the clinical study protocol was supported by the medical ethics committee of The Second Affiliated Hospital of Nanchang University.

### NAFLD model

Two weeks old C57BL/6 mice were purchased from Charles River (Beijing, China), and fed with HFD (MD12032, Medicinece, Jiangsu, China) for 16 weeks to make the NAFLD model. Subsequently, diethylnitrosamine (DEN, N0258, Sigma, St. Louis, MO) with 45 mg/kg *i.p*. in PBS was injected into NAFLD mouse model via intraperitoneal. All liver tissues were separated at 8, 16 and 36 weeks later for HFD feeding and 20 weeks later for DEN treatment, separately. The animal studies were approved by the Institutional Animal Care and Use Committee of the Second Affiliated Hospital of Nanchang University.

### NAFLD-HCC model

NAFLD-HCC mouse model was induced as pervious [17]. 45 mg/kg DEN was injected into 2 weeks old C57BL/6 mice by intraperitoneal injection. Then, the mice were randomly fed with HFD or normal diet (Medicinece, Jiangsu, China) at their 8 weeks old, and the feeding method was continued 10 weeks. Meanwhile, at their 8 weeks old, 5 × 10^7^ transducing units of lentiviruses encoding pcDNA-FTX (LV-FTX) in PBS or its negative control (LV-NC) together with 10 μg/g gadolinium III (GdCl_3_, 10138-52-0, Sigma) were used to treat the HCC mouse via tail vein injection.

### Cell culture and treatment

Liver tissue was perfused with 37 ℃ D-Hanks solution (Gibco, Grand Island, NY) through portal vein, and then D-Hanks was replaced with 37 ℃ 0.5 mg/ml collagenase IV (Sigma) in Dulbecco’s Modified Eagle Medium (DMEM, Gibco) when the color of liver changed from dark red to khaki. At 5 min later, separating the liver and cutting it into 1 mm pieces in 5 ml collagenase IV solution, and then the pieces were digested for 15 min at 37 ℃. Subsequently, the cell suspension was crushed though a 70 μm cell strainer followed by centrifuging for twice at 4 ℃ with a condition of 68 g centrifugal force and 5 min. Next, one centrifugation cycle at 1400 g for 30 min was performed to purify the non-parenchymal cells in an 8.2–17.6% Optiprep gradient. All cells at the interface were collected, and maintained with anti-CD11b microbeads (Miltenyi Biotec, Bergisch Gladbach, Germany) for 15 min followed by separating using the LS MACS columns (Miltenyi Biotec). At last, immunofluorescence assay was used to ensure the KCs though labeling F4/80. FTX siRNA and its scramble sequences were transfected into KCs stimulated with 0.25 mmol free fatty acid (FFA), respectively, using Lipofectamine 2000 (Invitrogen, Carlsbad, CA).

### Quantitative RT-PCR (qRT-PCR) assay

Total RNAs were extracted from human liver tissues, animal liver tissues or KCs using TRIzol reagent (Invitrogen), and then reverse-transcripted into cDNA employing the SuperScript One-Cycle cDNA Kit (Qiagen, Hilden, Germany). For quantitative PCR assay, the amplification was measured using a SYBR Premix Ex Taq kit (Takara Bio, Shiga, Japan) on Applied Biosystems 7500 Fast Real-Time PCR system (Applied Biosystems, Foster City, CA, USA). The primer sequences we used as follows: lncRNA FTX: 5′-GTGTCTCTCTCTCTCTCTCTCTT-3′ (forward), 5′-CCTCTTCAG CAGTAGCATAGTT-3′ (reverse); TNFα: 5′-CTGCCTGCTGCACTTTGGAG-3′ (forward); 5′-ACATGGGCTACAGGCTTGTCACT-3′ (reverse); iNOS: 5′-CTCACTG GGACAGCACAGAA-3′ (forward), 5′-TGGTCAAACTCTTGGGGTT C-3′ (reverse); IL-10: 5′-TGCCTTCAGCAGAGTGAAGA-3′ (forward), 5′-GTCTTGGTTCTCAGC TTGGG-3′ (reverse); CD206: 5′-TTCGGTGGACTGTGGACGAGCA-3′ (forward); 5′-ATAAGCCACCTGCCACTCCGGT-3′ (reverse); Arg-1: 5′-CATATCTGCCAAAG ACATCGTG-3′ (forward), 5′-GACATCAAAGCTCAGGTGAATC-3′ (reversed); GAPDH: 5′-ACCACAGTCCATGCCATCAC-3′ (forward), 5′-TCCACCACCCTGTT GCTGTA-3′ (reverse). FTX and mRNA were normalized to GAPDH. All reactions were repeated three times, and the data were calculated using the 2^−∆∆Ct^ method. All operates were carried out according to the manufacturer’s instructions.

### Immunohistochemistry assay

Human liver tissues were obtained and embedded using paraffin, and then were cutted into 4 μm slices. Next, all slices were conventionally dewaxed, and incubated with 100 ℃ citrate buffer (pH 6.0) for 40 min. Then, 10 min later for maintaining with 3% H_2_O_2_ at room temperature, the slices were incubated with primary antibodies against iNOS (Abcam, Cambridge, MA) and CD206 (Abcam) at 4 ℃ for overnight. Subsequently, the slices were incubated with secondary HRP-conjugated antibody (Abcam) for 1 h, fresh diaminobenzidine (Merck, Whitehouse Station, NJ) solution for 5 min, and hematoxylin for 3 min. At last, all slices were observed under a Leica microscope.

### Flow cytometry assay

Flow-cytometry assay were implemented to ensure the percentage of M1 and M2 KCs in liver tissues. The liver tissues were isolated, grind and centrifuged with a 3000 r/min speed for 10 min. Next, the precipitation were incubated with collagenase IV and DNase I solution, and then the liver cells were obtained though density gradient centrifugation. Subsequently, the cells were stained with FITC-iNOS and PE-CD68, and the fluorescence intensity was measured by a Flow Cytometer Cyan (Beckman Coulter).

### H&E staining assay

Hematoxylin–Eosin/HE staining kit (Solarbio, Shanghai, China) was used to display the pathological change of liver tissues in animal. Liver sections were stained using hematoxylin solution for 5–10 min after dewaxed and hydrated, and then combined with 1% hydrochloric acid ethanol for 30 s. Double distilled water was used to wash the slices which then were stained using the eosin solution for suitable time. Next, the slices were washed, dehydrated, sealed and subsequent observed.

### Detect the levels of ALT and AST

The production of alanine aminotransferase (ALT) and aspartate aminotransferase (AST) were measured by enzymatic colorimetric methods.

### Immunofluorescence assay

KCs were fixed by pre-cooled 4% paraformaldehyde for 30 min, infiltrated by 0.1% Triton X-100 for 10 min, blocked by 1% BSA (Sigma) for 30 min, incubated with primary antibody against F4/80 (Abcam) at 4 ℃ overnight, and maintained with rhodamine-conjugated goat anti-rabbit antibody (Invitrogen) for 30 min, successively. Next, all cells were stained by DAPI (Invitrogen), and photographed using a confocal microscope.

### ELISA assay

ELISA assay was carried out to ensure the levels of TNF-α and IL-10 in cell culture supernatant according to the manufacturer’s introductions. TNF-α and IL-10 ELISA kits were purchased from eBioscience (San Diego, Calif).

### Data analysis

All analyses were performed though SPSS v12.0 software, and all data are presented as mean ± standard deviation (SD). The difference of two groups was ensured by student’s *t* test, and one-way analysis of variance was used for multiple groups. *p*-values of less than 0.05 was considered as a statistically significant. All data were ensured by the experiments which repeated at least three times.

## Results

### FTX expression and M1/M2 KCs ratio were decreased in NAFLD-HCC

**Fig. 1:**
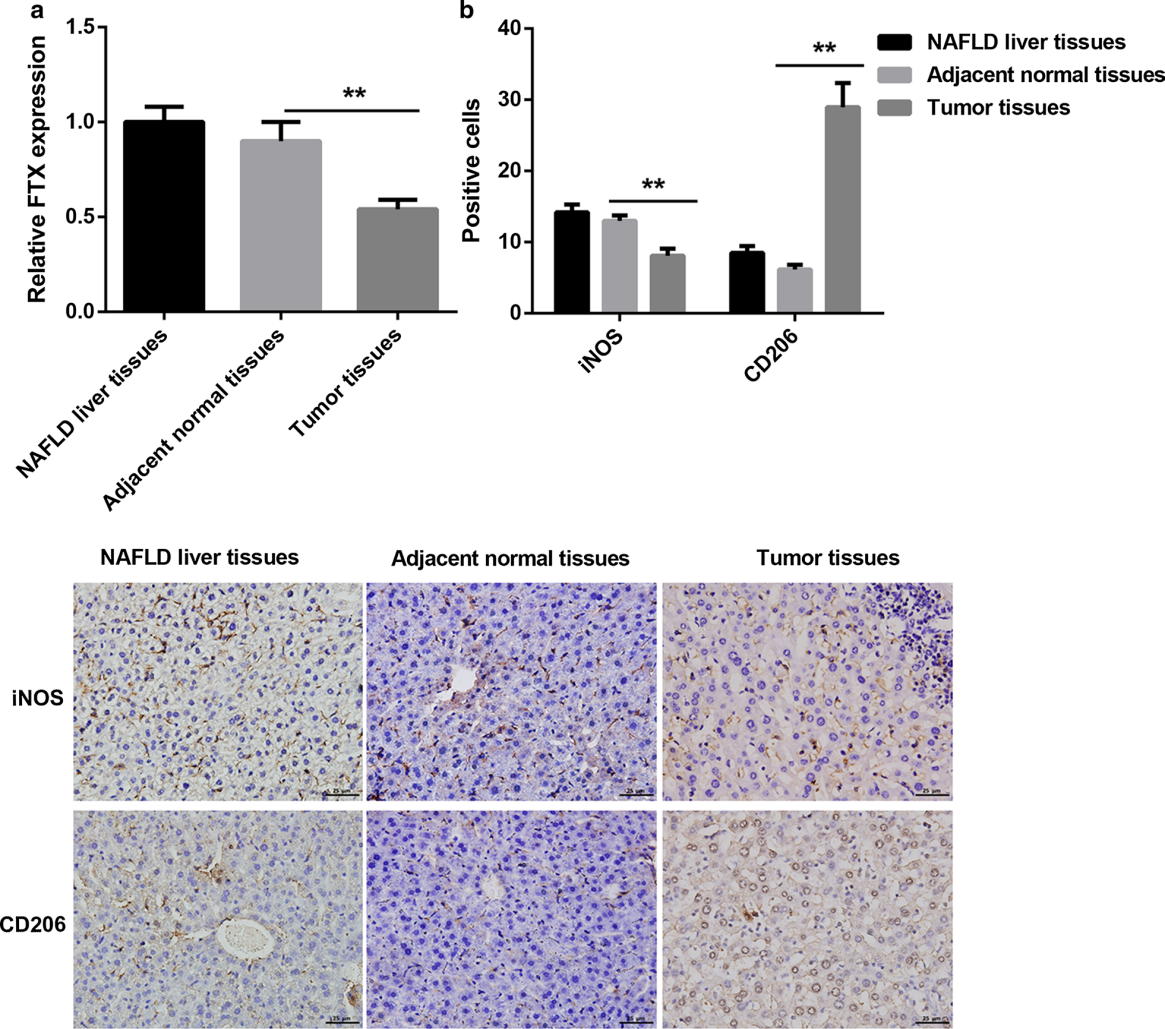
The expression of FTX and the number of M1 polarized KCs were decreased in NAFLD-HCC. **a** The expression of FTX was measured by qRT-PCR assay. **b** The cell number of iNOS-positive and CD206-positive were ensured by immunohistochemistry. Here, iNOS is one of markers of M1 polarized KCs and CD206 is one of markers of K2 polarized KCs. **p < 0.01

**Fig. 2:**
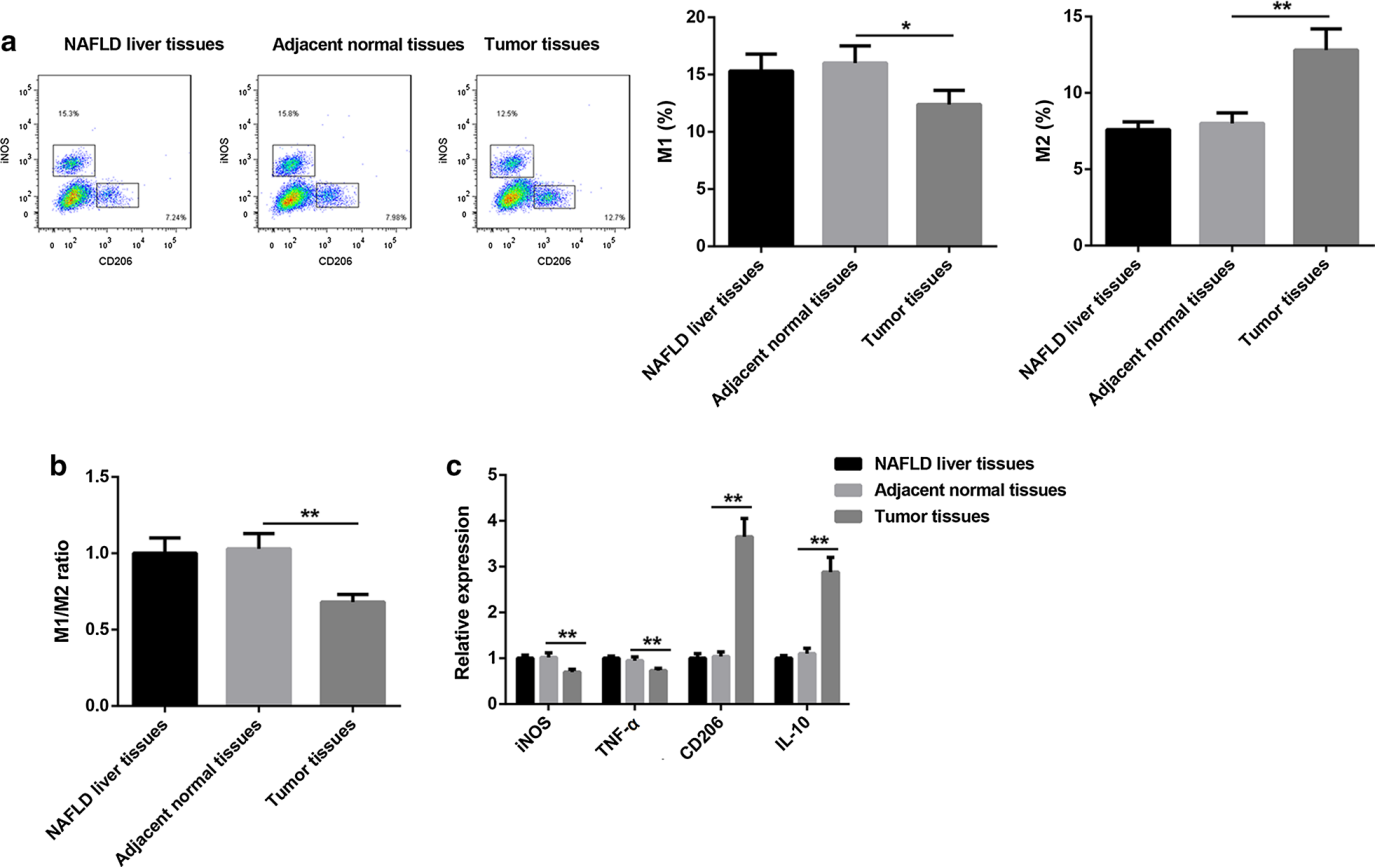
M1 polarization of KCs was repressed in NAFLD-HCC, but M2 polarization of KCs was promoted. **a** Flow cytometry assay was fulfilled to measure the percentage of M1 and M2 KCs. **b** The ratio of M1/M2 KCs was analyzed. **c** The expression of iNOS, TNF-α, CD206 and IL-10 mRNAs were detected using qRT-PCR. Here, iNOS and TNF-α were used to represent M1 KCs, and CD206 and IL-10 were used to represent M2 KCs. *p < 0.05, **p < 0.01

Firstly, we ensured the expression of FTX and the percentage of M1 and M2 KCs in diseased liver tissues from NAFLD and NAFLD-HCC patients and adjacent normal tissues from NAFLD-HCC patients. As shown in Fig. 1a, the expression of FTX was significantly decreased in tumor tissues. Moreover, the immunohistochemistry results indicated that the number of iNOS-positive cells was downregulated in tumor tissues, while CD206-positive cells was increased (Fig. 1b). Consistently, the results of flow cytometry analysis displayed that the percentage of M1 KCs was downregulated in tumor tissues, while M2 KCs was upregulated (Fig. 2a). The ratio of M1/M2 KCs was notably decreased in tumor tissues (Fig. 2b). Moreover, we further found that the expression of iNOS mRNA and TNF-α mRNA were decreased in tumor tissues, while CD206 mRNA and IL-10 mRNA were increased (Fig. 2c). All in all, our data demonstrated that the expression of FTX and the ratio of M1/M2 KCs were decreased in tumor tissues of NAFLD-HCC.

### FTX expression and M1/M2 KCs ratio were decreased during NAFLD conversion to HCC

**Fig. 3:**
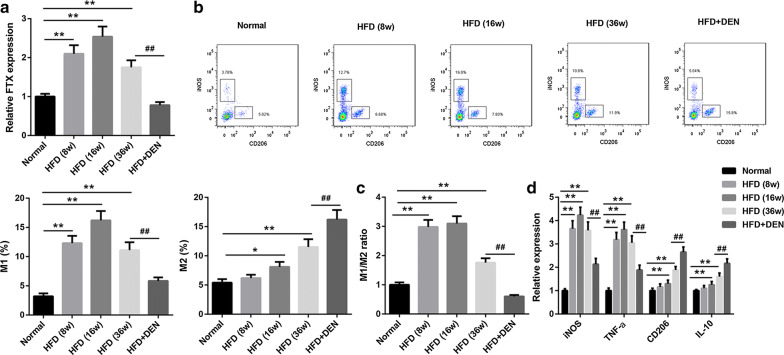
FTX expression and the value of M1/M2 KCs were reduced during NAFLD conversion to HCC. **a** qRT-PCR assay was applied to detect the expression of FTX in liver tissues from each animals. **b**, **c** The percentage of M1 and M2 KCs were ensured by flow cytometry and analyzed. **d** The expression of iNOS, TNF-α, CD206 and IL-10 mRNAs were measured by qRT-PCR. *p < 0.05, **p < 0.01 and ^#*#*^*p* < 0.01

To investigate the variation of FTX expression and M1/M2 KCs ratio during NFLAD conversion to HCC, we established NAFLD mouse model using HFD. After treated with HFD for 8, 16 and 36 weeks, and DEN for 20 week, all animals were euthanized, and then the liver tissues were isolated for next experiments. Our results indicated that the expression of FTX was increased in HFD groups, while inhibited by DEN (Fig. 3a). Hence, we thought that the expression of FTX was downregulated during NAFLD to HCC conversion. Besides, as shown in Fig. 3b, the percentage of M1 KCs in liver tissues was notably upregulated in HFD group, but reduced after DEN treatment. Oppositely, M2 KCs percentage was upregulated in HFD group, and facilitated by DEN treatment. HFD resulted in the upregulation of M1/M2 kCs ratio, while DEN combined HFD to induce the decreasing of M1/M2 KCs ratio (Fig. 3c). Next, we further displayed that HFD upregulated the expression of iNOS mRNA, TNF-α mRNA, CD206 mRNA and IL-10 mRNA, among these factors, the expression of iNOS mRNA and TNF-α mRNA were inhibited by DEN, however, CD206 mRNA and IL-10 mRNA expression were further upregulated by DEN (Fig. 3d). Overall, the ratio of M1/M2 KCs was increased in NAFLD compared with normal, while it was decreased in the process of NAFLD to HCC conversion.

### FTX repressed the progression of NAFLD to HCC

**Fig. 4:**
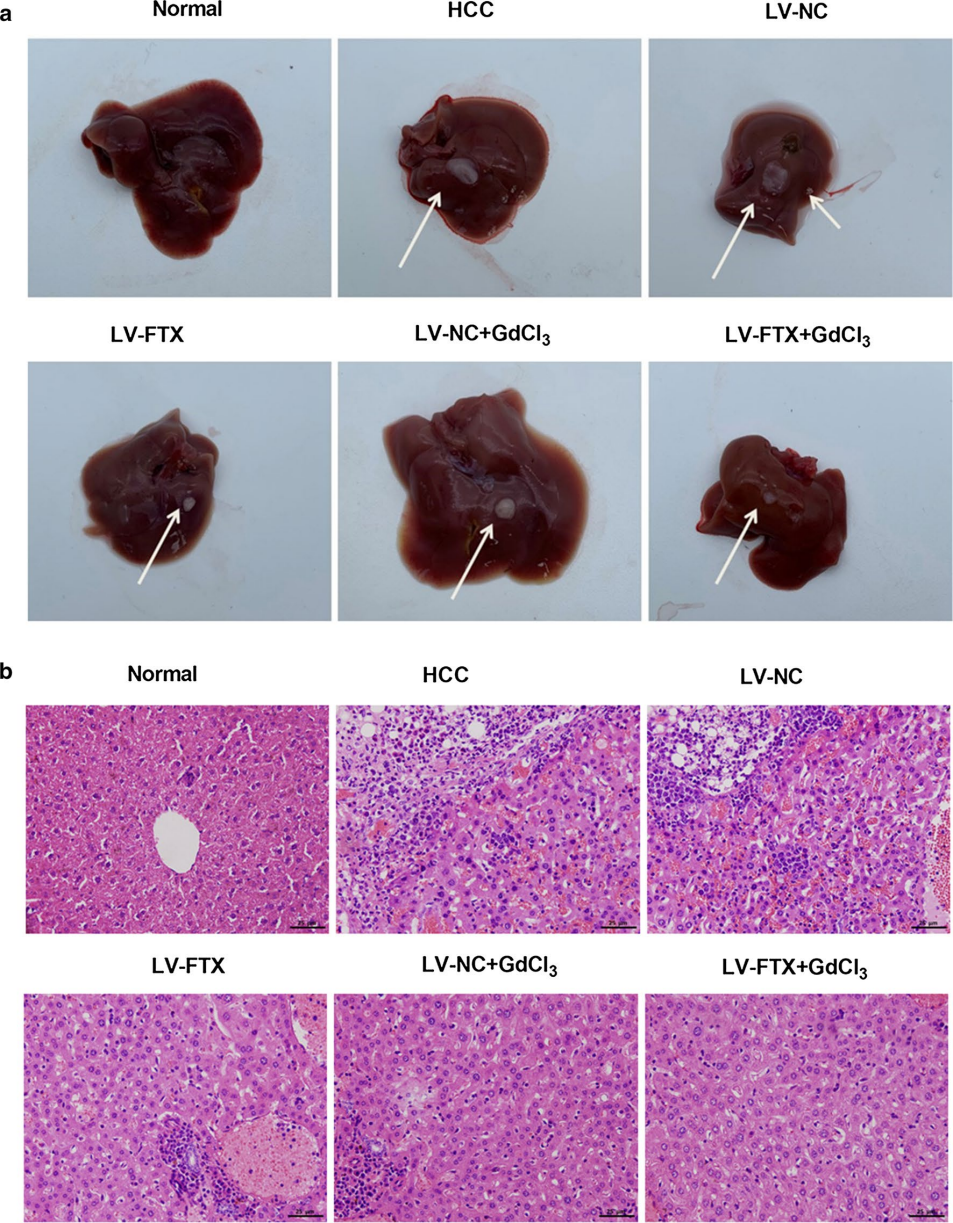
FTX increasing limited tumor formation and liver damage during NAFLD conversion to HCC. **a** All liver tissues were isolated and the tumors formation on it were showed. Arrow indicated the formed tumor. **b** HE staining was performed to measure the pathological change in liver tissues

**Fig. 5:**
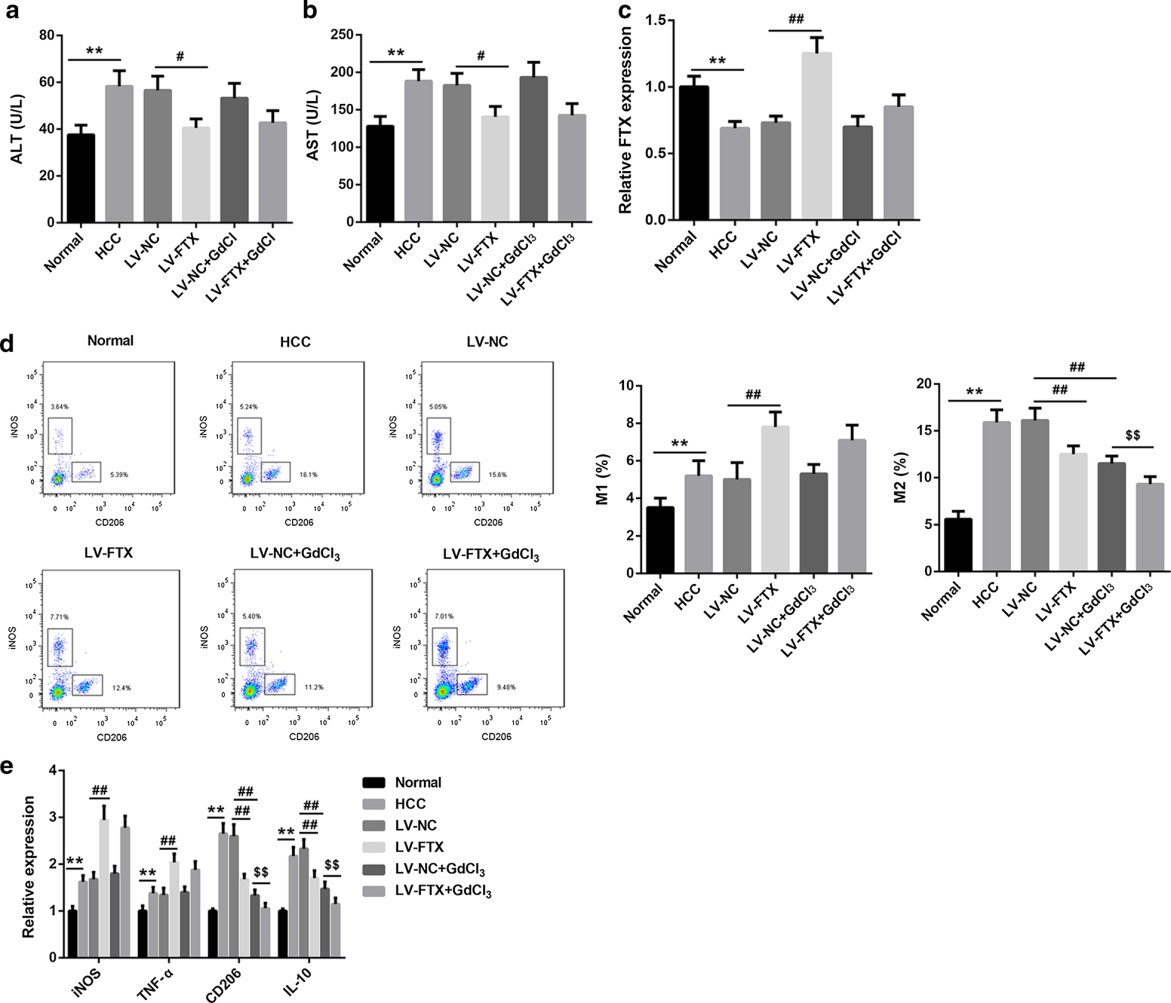
FTX increasing repressed NAFLD conversion to HCC via activating M1 polarization of KCs. **a**, **b** The production of ALT and AST in serum were measured by enzymatic colorimetric methods. **c** The expression of FTX in liver tissues was examined using qRT-PCR. **d** The percentage of M1 and M2 KCs were ensured using the flow cytometry. **e** The expression of iNOS, TNF-α, CD206 and IL-10 mRNAs were detected by qRT-PCR. **p < 0.01, ^#^*p* < 0.05, ^##^*p* < 0.01 and ^$$^*p* < 0.01

It has been demonstrated that GdCl_3_ could effectively induce the inactivation of KCs [18]. Here, to explore the effect of FTX on the conversion of NAFLD to HCC, LV-FTX with or without GdCl_3_ were used to treat the animals fed with HFD at 6 weeks after injection of 45 mg/kg DEN. The liver tissues were isolated from each animals after 10 weeks later of continuing HFD feed. As shown in Fig. 4a, the tumors formation were significantly suppressed by FTX increasing, while GdCl_3_ treatment could reverse the inhibitory effect of FTX increasing on tumor formation. The H&E staining results indicated that overexpression of FTX notably improved the injury of liver tissues induced by NAFLD-HCC, however, GdCl_3_ treatment repressed FTX increasing-induced the improvement of liver damage (Fig. 4b). Increasing of FTX limited NAFLD conversion to HCC through targeting KCs activations. Besides, the production of liver function indicator, ALT and AST, in serum was measured, the results shown that ALT and AST were upregulated in HCC group, and increasing of FTX could inhibit the production of ALT and AST (Fig. 5a, b). Furthermore, the expression of FTX was significantly downregulated in liver tissues from NAFLD-HCC mouse, but lentivirus infection effectively increased the expression of FTX (Fig. 5c). The flow cytometry data displayed that the percentage of M1 and M2 KCs were upregulated in HCC group, and overexpression of FTX significantly increased M1 and decreased M2 KCs. Meanwhile, GdCl_3_ treatment exacerbated the decreasing of M2 KCs induced by FTX overexpression (Fig. 5d). The genes expression of iNOS, TNF-α, CD206 and IL-10 also were promoted in HCC groups, and increasing of FTX facilitated iNOS and TNF-α genes expression, while repressed CD206 and IL-10 genes expression. Importantly, regardless of whether FTX increasing or not, GdCl_3_ treatment decreased the expression of CD206 and IL-10 genes (Fig. 5e). Overall, overexpression of FTX significantly inhibited the growth of tumor and improved the liver damage during NAFLD conversion to HCC via promoting KCs polarization to M1 phenotype.

### Decreasing of FTX promoted M2 polarization of KCs

**Fig. 6:**
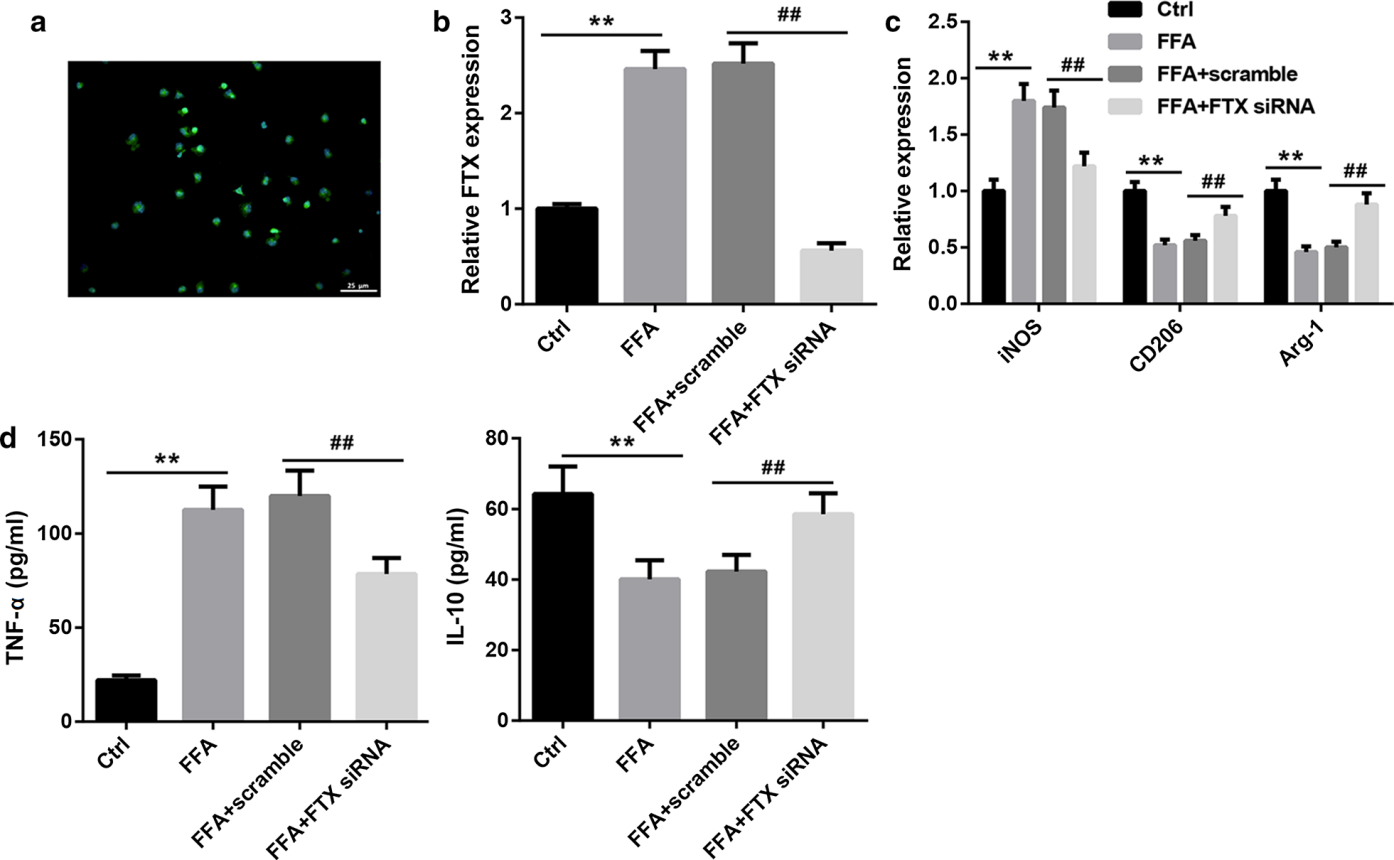
FTX increased M1 polarized KCs. **a** The primary KCs were ensured by immunofluorescence assay labeled F4/80. **b** The expression of FTX was measured using qRT-PCR. **c** The expression of iNOS, CD206 and Arg-1 mRNAs in KCs were measured by qRT-PCR. **d** The production of TNF-α and IL-10 in cell culture supernatant were detected using ELISA assay. **p < 0.01, ^##^*p* < 0.01 and ^#^*p* < 0.05

To ensure the role of FTX in KCs polarization, we used FFA to induce the primary KCs transfected with FTX siRNA or its scramble sequence. Firstly, we identified the primary KCs though immunofluorescence assay, and the results displayed that we obtained the KCs successfully (Fig. 6a). Meanwhile, FFA increased the expression of FTX in KCs, while limited by FTX siRNA (Fig. 6b). Next, we detected the genes expression of iNOS, CD206 and Arg-1. As shown in Fig. 6c, FFA leaded to the increase in iNOS mRNA and decrease in CD206 mRNA and Arg-1 mRNA, while their expression obviously reversed by FTX silencing. Furthermore, our data demonstrated that FFA promoted the production of TNF-α and inhibited IL-10, however, silencing of FTX could repress FAA-induced TNF-α increasing and IL-10 decreasing (Fig. 6d). Altogether, FTX promoted M1 polarization of KCs and FTX silencing significantly promoted KCs polarization to M2 phenotype.

## Discussion

Primary liver cancer is the second most common reason of cancer mortality worldwide. HCC accounts for more than 90% of all cases of primary liver cancer, and represents a major health problem [19]. With the research of HCC, NAFLD has been considered as the major contributor to HCC formation. While the precise mechanism during NAFLD conversion to HCC is unclear [20]. Accumulating reports have shown that altered lncRNAs play a curial role in the progression of HCC. For instance, the expression small nucleolar RNA host gene 16 (SNHG16) has been proved to be increased in tumor tissues and cell lines of HCC, and inhibition the expression of SNHG16 could repress the sorafenib resistance, proliferation, migration and invasion of HCC cell lines [21]. Another lncRNA, AWPPH, has been demonstrated to be upregulated in HCC, and increased AWPPH promotes the growth and metastasis of tumor [22]. Besides, a growing reliable studies have demonstrated that the development of NAFLD also regulated by a series of lncRNAs like NEAT1. It has been indicated that NEAT1 is upregulated in NAFLD mouse model, and inhibition the expression of NEAT1 could effectively inhibit the progression of NAFLD [23]. However, rare lncRNA have been reported during NAFLD to HCC conversion, the role and mechanism of action of lncRNAs in the conversion of NAFLD to HCC has not be fully understood. In this present study, we certificated that the expression of FTX in human NAFLD-HCC tissues was downregulated. Interestingly, we found that the expression of FTX was upregulated in NAFLD animals liver tissues, but was decreased in NAFLD-HCC animals liver tissues. Hence, we considered that the expression of FTX was downregulated during the conversion of NAFLD to HCC. Based on this conclusion, we asked that whether this change of FTX expression associated with the conversion of NAFLD to HCC. Next, we revealed that increasing of FTX effectively repressed the growth of tumor and improved the liver damage in NAFLD-HCC, thus to inhibit the progression of NAFLD to HCC. Interestingly, we further found that FTX inhibited the conversion through targeting KCs activation.

Liver is a complex organ which consisted by a series of cells such as KCs. They are the resident macrophage and account for about 20–25% of non-parenchymal cells in liver. KCs play an important role in the innate immune response [24]. Accumulation researches have indicated that KCs polarization is a crucial regulator to inflammatory response. It has been demonstrated that long-term HFD could induce the increasing of M1 polarization of KCs and promote the expression of pro-inflammatory such as TNF-α, balancing the percentage of M1 and M2 KCs could prevent the development of NAFLD [25]. However, how about the role of KCs polarization in the conversion of NAFLD to HCC remains unclear. Recently, Wang et al. demonstrated that the ratio of M1/M2 KCs in NAFLD-HCC tissues was downregulated, silencing the expression of SNHG20 could delay the progression of NAFLD to HCC via regulating the polarization in macrophages [10]. This study demonstrated that lncRNA may regulate the progression of NAFLD to HCC by targeting to the polarization of KCs. In this present study, we demonstrated that the values of M1/M2 KCs was downregulated in the process of NAFLD to HCC conversion, and increasing the expression of FTX could promote M1 polarization of KCs, thus to limit this conversion.

## Conclusion

In summaries, our data revealed that the expression of FTX was downregulated during NAFLD conversion to HCC, and upregulation of FTX inhibited the conversion of NAFLD to HCC via promoting KCs polarization to M1 phenotype. We pointed out FTX is an effective therapeutic target for NAFLD-HCC. Our data proposed a new regulatory mechanism of NAFLD conversion to HCC, and supplemented our scanty knowledge on the role of lncRNA in NAFLD conversion to HCC. However, more researches needed to be carried out to explore the action mechanism of FTX in the conversion of NAFLD to HCC. For instance, whether FTX could repress the conversion through acting as a miRNA sponge or a RNA-binding protein. Which molecules and mechanisms involve in regulating the expression of FTX in this conversion. All these questions needed to be further investigated.

## Data Availability

The datasets used and/or analyzed during the current study are available from the corresponding author on reasonable request.

## Abbreviations

HCC: Hepatocellular carcinoma
HFD: High fat diet
KC: Kupffer cell
LncRNAs: Long non-coding RNAs
NAFLD: Non-alcoholic fatty liver disease
NAFLD-HCC: NAFLD-transformed HCC
